# Stressful Dieting: Nutritional Conditions but Not Compensatory Growth Elevate Corticosterone Levels in Zebra Finch Nestlings and Fledglings

**DOI:** 10.1371/journal.pone.0012930

**Published:** 2010-09-28

**Authors:** Mariam Honarmand, Wolfgang Goymann, Marc Naguib

**Affiliations:** 1 Department of Animal Behaviour, University Bielefeld, Bielefeld, Germany; 2 Department of Animal Behaviour, Freie Universität Berlin, Berlin, Germany; 3 Max-Planck-Institut für Ornithologie, Abteilung für Verhaltensneurobiologie, Evolutionary and Environmental Physiology Lab, Seewiesen, Germany; 4 Department of Animal Ecology, Netherlands Institute of Ecology (NIOO-KNAW), Heteren, The Netherlands; Stockholm University, Sweden

## Abstract

Unfavourable conditions throughout the period of parental care can severely affect growth, reproductive performance, and survival. Yet, individuals may be affected differently, depending on the developmental period during which constraints are experienced. Here we tested whether the nestling phase compared to the fledgling phase is more susceptible to nutritional stress by considering biometry, physiology, sexually selected male ornaments and survival using zebra finches (*Taeniopygia guttata*) as a model species. As nestlings (day 0–17) or fledglings (day 17–35), subjects were raised either on low or high quality food. A low quality diet resulted in significantly elevated baseline corticosterone titres in both nestlings and fledglings. Subjects showed substantial compensatory growth after they had experienced low quality food as nestlings but catch-up growth did neither lead to elevated baseline corticosterone titres nor did we detect long term effects on biometry, male cheek patch, or survival. The compensation for temporally unfavourable environmental conditions reflects substantial phenotypic plasticity and the results show that costs of catch-up growth were not mediated via corticosterone as a physiological correlate of allostatic load. These findings provide new insights into the mechanisms and plasticity with which animals respond to periods of constraints during development as they may occur in a mistiming of breeding.

## Introduction

A key period for vertebrates is early in life when biosynthetic and growth rates are steeper than later in life requiring a high energy and adequate nutrient intake. Among vertebrates, birds have been a key model for investigating effects of environmental conditions during early development [1]–[6]. Birds, unlike mammals, cannot use own body resources to buffer their offspring against even short periods of poor nutritional conditions. An altricial chick, depending entirely on parental care, can face serious problems when environmental conditions fluctuate or decline. As a consequence, reproductive decisions and breeding should be timed with respect to periods of high food availability to match the period of maximum offspring growth and development. Yet, optimal timing can be difficult, because fluctuating nutritional conditions, transient changes in climate, or predator and competitor abundance are not entirely predictable [7], [8]. For instance, reproducing early in the season may allow parents to raise a second brood [9], [10] but it may also bear the risk of low food availability during early chick rearing. In contrast, breeding late in the season likely results in the opposite scenario where a period of high food availability during the nestling phase (nestlings stay in the nest and are entirely dependent of parental care) is followed by a period of declining food abundance during the fledgling phase (fledglings leave the nest, start feeding by themselves but are not yet independent of parental care). As growth trajectories and physiological demands differ substantially between nestlings and fledglings, the ecological conditions experienced during either phase are likely to differentially shape the phenotype. Yet, there is little experimental evidence comparing effects of nutritional shortage during these stages. In most studies showing pronounced phenotypic effects, subjects were exposed to a stressor either throughout the entire period of parental care [2], [4], [11], [12] or only during the nestling period [13], [14]. Two studies recently showed that adult birds which had experienced nutritional stress during the nestling period rather than the fledgling period were less able to compensate in terms of management of energy resources (resting metabolic rate and body mass loss under deprivation) [15], [16].

Such long-term effects may well be linked to immediate physiological stress responses during the period of food restriction. Resource limitations can impose energetic stress for a developing vertebrate and can via activation of the hypothalamic-pituitary-axis (HPA) increase glucocorticoid secretion [17], [18]. Glucocorticoids have been suggested to play an important role when an organism copes with unpredictable environmental events. During such events glucocorticoids mediate (and indicate) the increasing costs of maintaining allostasis [19], [20] or physiological stability [21].

Short-term elevations of glucocorticoids can be adaptive because they stimulate immune function, cognitive performance and free energy resources [22], [23]. Even though glucocorticoid secretion following a stressor allows an adequate behavioural and physiological response to ensure survival, chronic elevations of glucocorticoids resulting in inhibition of anabolic processes such as growth may become detrimental [23].

At an early age, the balance between costs and benefits of glucocorticoid's action additionally depends on the state of maturation at hatching/birth according to different life history strategies (slow vs. fast maturation or precocial vs. altricial). Slow strategists and precocial species hatch or are born at a more mature state and are more independent from their parents than fast strategists and altricial species [24]. Consequently, precocial young can actively change their foraging behaviour or reduce exposure to risks in contrast to altricial young, which are nest bound and entirely dependent of parental care. Altricial nestlings can increase begging behaviour and aggressiveness towards their siblings to improve energy intake and they may allocate resources to important processes in an hierarchical order [25], [26], but until the capacities of behavioural responses to challenges develop (e.g. around fledging) increased glucocorticoid secretion appears to be little beneficial. Experimental evidence for an up regulation in glucocorticoid titres as a response to stressful conditions in nestlings is mixed ([18], [28] but [27]), even though some studies suggest that altricial nestlings may indeed exhibit adequate physiological and behavioural adjustments in response to stressful situations [29], [30]. Therefore, it remains unclear whether a demanding period such as unfavourable environmental conditions or the extra energetic demand incurred to support catch-up growth, results in an elevation of corticosterone levels in young altricial birds.

**Figure 1:**
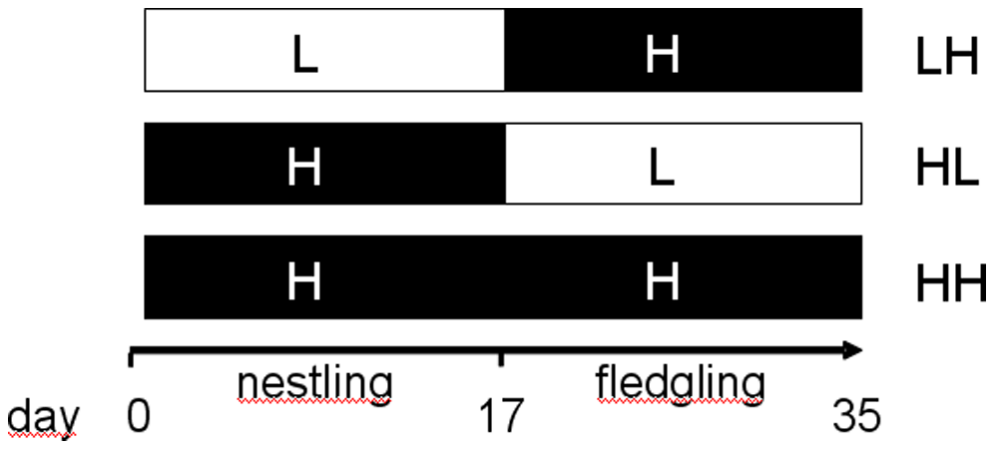
Overview of experimental treatments and time course. The three experimental groups received a low (L) or a high (H) quality rearing diet in different order during their first month post hatching. The low-high (LH) group received low quality food from day 3 until day 17 and high quality food from day 17 until day 35 ( = breeding earlier than optimal). The high-low (HL) group received high quality food from day 3 until day 17 and low quality food from day 17 until day 35 (breeding later than optimal). The high-high (HH) group received high quality food from day 3 until day 35 (optimal timing of breeding).

Here we studied whether nestlings and fledglings are differentially susceptible to unfavourable nutritional conditions using zebra finches (*Taeniopygia guttata*) as model species. To test whether nestlings are more vulnerable to low quality nutrition than fledglings, we exposed breeding pairs during either developmental stage of their offspring (nestling or fledgling) to qualitatively different diets. Breeding pairs received the two diets (low [L-] and high [H-] quality) in different order to simulate three natural scenarios: breeding earlier than optimal (low-high; LH-group), breeding later than optimal (high-low; HL-group), and a positive reference for optimal timing (high-high; HH- group; see Fig. 1). We measured offspring biometry throughout development and determined baseline corticosterone levels as measure of allostatic load during periods of relatively poor nutritional condition and during periods of catch-up growth. Moreover, we quantified male cheek patch expression to reveal possible differences in the timing or quality of moulting sexually selected ornaments and analyzed whether on a long-term scale there was an effect on survival.

## Results

### Effects on biometry

**Figure 2:**
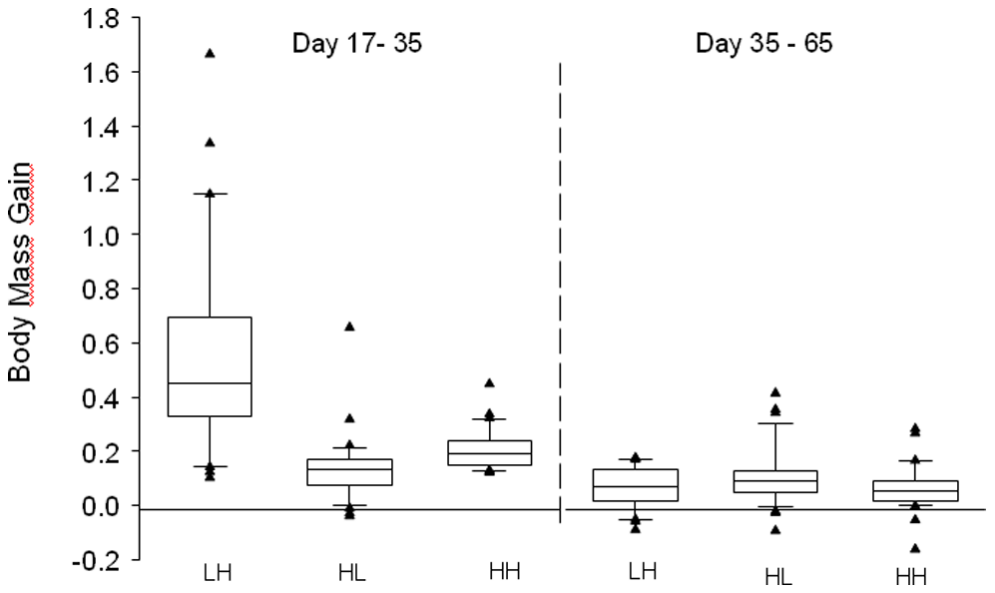
Body mass gain from day 17 to 35 and from day 35 to 65. Shown are medians ± quartiles for relative change in body mass (body mass 2 – body mass 1/body mass at hatching) across the three experimental treatment groups.

At day of hatching (d0), offspring body mass did not differ between treatment groups (LME: *F*_2,32_ = 0.03, *P* = 0.97). Subjects raised on a high quality diet (H) grew faster than subjects on a low quality diet (L) resulting in significant differences in all biometric traits at day 17 and at day 35 on wing length (Table 1). At day 35 there was a trend for females to have lower mean body mass and shorter tarsi than males when dieting either as nestlings (LH) or fledglings (HL) (Table 1 and Table S1 in supplement). There was a significant treatment effect on catch-up growth from day 17 until 35 (Kruskal-Wallis: *Chi^2^* = 43.51, n = 96, df = 2, *P*<0.001; Fig. 2) whereas there was no such effect from day 35 until day 65 (Kruskal-Wallis: *Chi* ^2^ = 3.60, n = 96, df = 2, *P* = 0.17; Fig. 2). Offspring which had experienced low quality nutrition either as nestlings (LH) or fledglings (HL) had compensated in body mass and tarsus length by day 65 but the effect on wing length persisted until day 280 (Table 1, Table S1 in supplement).

**Table 1:** Effect of nutrional treatment on biometry and baseline corticosterone levels.

	Day 17			Day 35		Day 65		Day 280	
	N = 33			n = 96, N = 33		n = 95, N = 33		n = 89, N = 33	
trait	n	F_1,31_	p	F_2,30_	p	F_2,30_	p	F_2,30_	p
body mass	99	31.3	<0.001	2.02	0.15^1^	1.57	0.23^3^	2.42	0.11^4^
wing	98	6.55	0.016	4.54	0.02	9.78#	0.008	0.39	0.69^5^
tarsus	98	7.3	0.01	1.74	0.19^2^	0.40	0.68	0.72	0.49
		n = 71, N = 30		n = 67, N = 30					
corticosterone		F_1,23_ = 4.81	0.04^6^	F_2,27_ = 2.30	0.12^7^				

Effects tested with linear mixed effect models (LME) with upper case numbers indicating significant factors/interactions within the final model (N  =  nests, which subjects came from, n  =  subjects).

1 sex: F_1,25_ = 3.4, p = 0.079; treatment*sex: F_1,25_ = 3.04, p = 0.066.

2 sex: F_1,25_ = 2.72, p = 0.11; treatment*sex: F_1,25_ = 4.88, p = 0.016.

3 sex: F_1,23_ = 1.52, p = 0.70; treatment*sex: F_2,23_ = 4.09, p = 0.03.

4 sex: F_1,53_ = 0.085, p = 0.77; treatment*sex: F_2,53_ = 3.36, p = 0.04.

5 sex: F_1,55_ = 6.04, p = 0.017.

6 brood size: F_5,23_ = 2.42, p = 0.067; sampling order F_5,9_ = 7.16, p = 0.006.

7 sex: F_1,8_ = 24.55, p = 0.001; sampling order: F_3,8_ = 5.18, p = 0.028; sampling time: F_1,8_ = 10.74, p = 0.011.

# Kruskal Wallis Chi^2^.

### Effects on corticosterone

**Figure 3:**
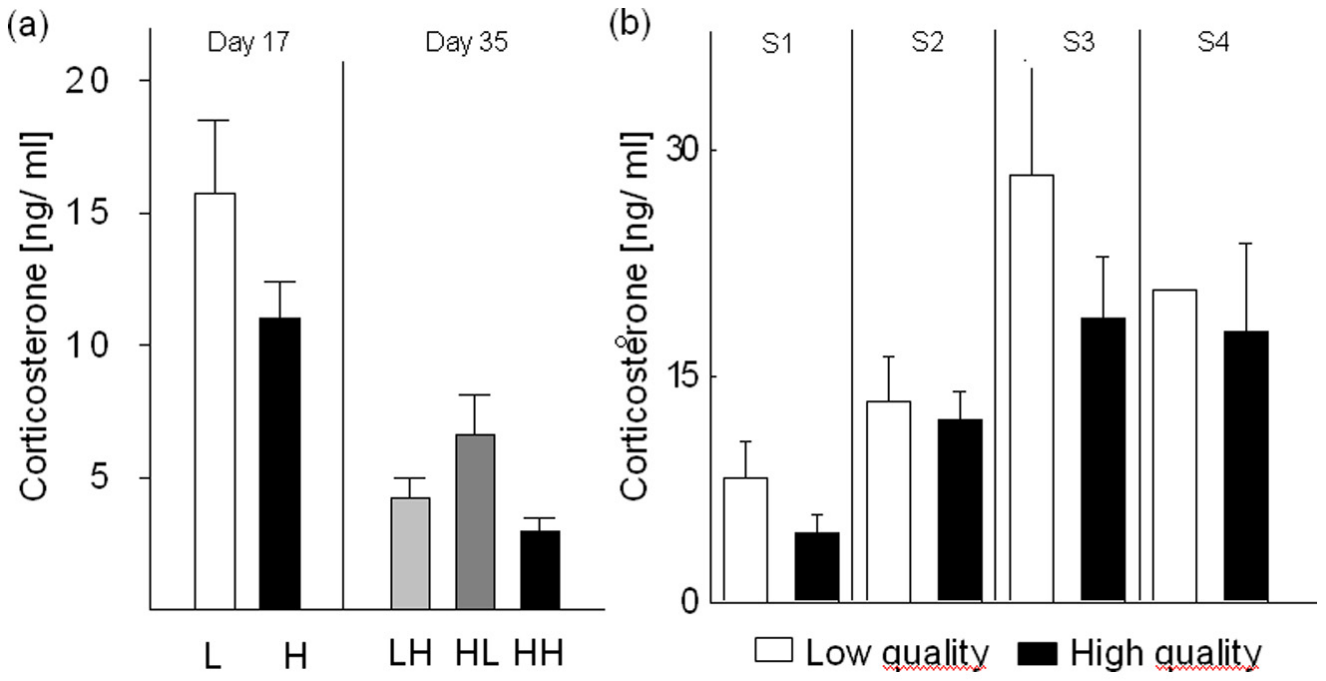
Corticosterone concentrations. a) Mean ± s.e corticosterone baseline titres at day 17 for subjects raised on low (L) or high (H) quality food and at day 35 when subjects experienced one of the three experimental treatments: low-high (LH), high-low (HL) or high-high (HH). Note: day35 only shows values within detection limits. b) Mean ± s.e. corticosterone baseline titres at day 17 across individual position within blood sampling sequence (S1–S4) for L- and H-subjects. As only one L- subject was sampled at the forth position (S4) s.e. do not apply.

Nutritional treatment resulted in significantly higher baseline corticosterone concentrations in the low quality group at day 17 (Table 1; Fig. 3 a, b) but there was no such effect at day 35 (Table 1). However, in 68% of the samples at day 35, the corticosterone concentration was below the detection limit (59% from LH, 55% from HL and 75% from HH), whereas this distribution was not significantly different across groups (Chi^2^ = 1.98, df = 2, p = 0.37). Therefore a second analysis on samples within detection limits was run (LH = 9, HL = 10, HH = 6) showing a significant treatment effect on baseline corticosterone levels at day 35 (LME: *F*_2,16_ = 3.93, *P* = 0.04; sex: *F*_1,1_ = 10.43, *P* = 0.19, sampling sequence: F_3,1_ = 3.46, *P* = 0.37; time: F_1,1_ = 7.28, *P* = 0.23; Fig. 3a). Parameter estimates of the final model (second analysis) revealed that baseline corticosterone from HL subjects was elevated compared to LH subjects (*P* = 0.003) and to HH subjects (*P* = 0.0013), whereas no difference between LH and HH subjects was detected (*P* = 0.65). There was an overall significant decrease in baseline corticosterone concentration from day 17 to day 35 (Wilcoxon: *W* = 3993, n_day17_ = 71, n_day35_ = 67, *P*<0.001; Fig. 3a).

### Effects on male plumage ornaments and survival

Male cheek patch development did not differ significantly between subjects raised in the different nutritional treatment groups (mean of cheek/bill ratio ±s.e.: LH_n = 15_ = 1.55±0.13, HL_n = 17_ = 1.39±0.39, HH_n = 12_ = 1.24±0.25; Kruskal-Wallis: *Chi* ^2^ = 2.32, n = 44, df = 2, *P* = 0.31). However, males raised under HH conditions tended to be more variable in their cheek patch size (LH range = 0.36–2.48, HL range = 0.19–2.38, HH = 0.16–3.02; Fisher: *F* = 2.97, df_num_ = 11, df_denom_ = 14, *P* = 0.059).

**Figure 4:**
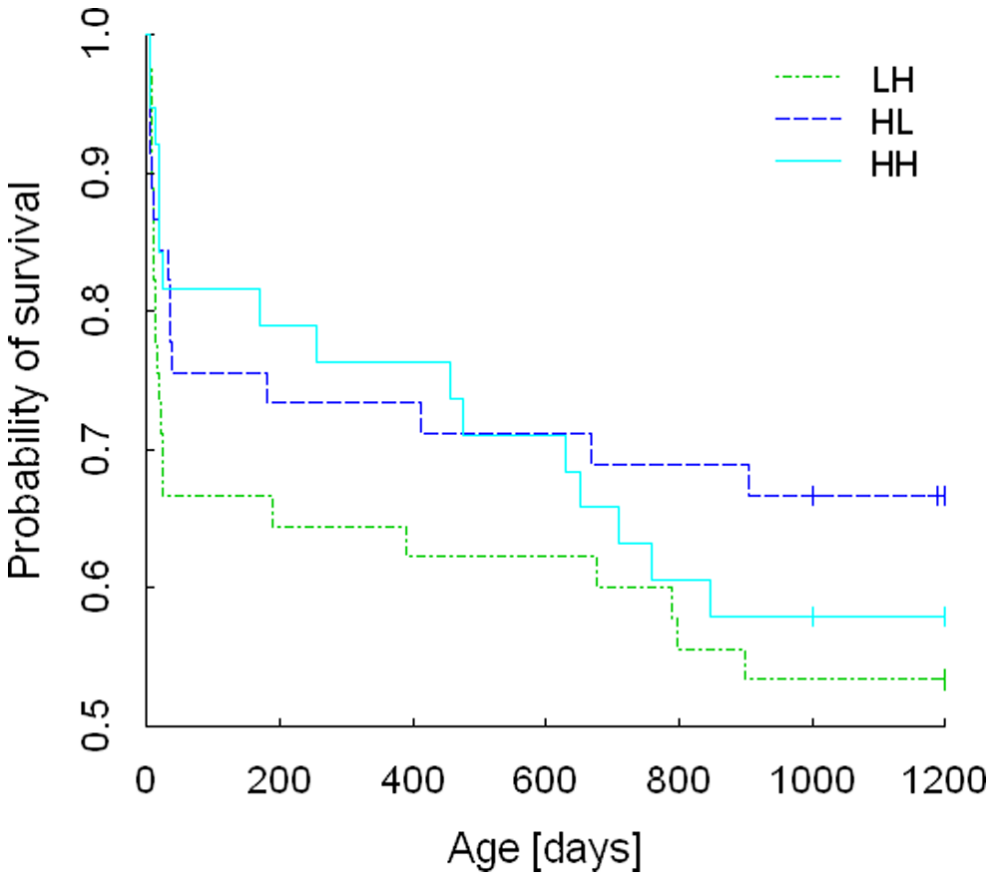
Survival Analysis (Kaplan-Meier). Probability of survival for the three treatment groups: low-high (LH), high-low (HL) or high-high (HH) with censoring at day 1200.

On a long-term scale, a higher proportion of females than males died (*Chi* ^2^ = 4.50, df = 1, *P* = 0.034), which was not explained by nutritional treatment (*Chi* ^2^ = 1.58, df = 2, *P* = 0.45; Fig. 4) even though a higher proportion of birds from the LH group (36%) died compared to 25% HL and 24% HH. During and shortly after the experimental manipulations there was no effect of sex on survival (LME: *F*_2,11_ = 0.86, *P* = 0.45; considering deaths from ages day 5 to 40).

## Discussion

The experiments revealed that nestling zebra finches were more susceptible to unfavourable nutritional conditions than fledglings. Despite fledglings being more robust against poor nutrition, baseline corticosterone was significantly elevated during a low quality rearing diet in both, nestlings and fledglings. Yet, nestlings had significantly higher corticosterone levels than fledglings. Even though birds raised on low quality food as nestlings (LH) showed remarkable catch-up growth, this resulted neither in elevated corticosterone levels during the catch-up growth period nor in long-term consequences on male plumage ornaments or survival.

Restricted periods of low food supply are common in nature and may occur at a similar frequency as longer periods of low quality food applied by many studies [4], [10], [11]. Our findings of differential responses by nestlings and fledglings to diets differing in quality thus expand on previous studies and have implications for understanding adaptations of opportunistic and seasonal breeders to fluctuations in food supply. Apparently, periods of low quality food carry more costs if experienced as nestlings than as fledglings and the resulting catch-up growth can cause long-term effects [6], [16]. This indicates that breeding too early can carry more costs for the offspring than breeding too late, but that substantial plasticity allows for phenotypic compensation when constraints are experienced during only part of the developmental period. These effects may have played a role in the evolution of strategies to refrain from initiating reproduction too early within the season. They could provide one possible explanation for the observed mistiming of reproduction in species where food peaks have moved forward due to clime change and for which birds did not fully compensate in terms of laying dates [8]. This may be a general mechanism, even though granivorous, opportunistic breeders, as zebra finches, do certainly face different challenges than insectivorous, seasonal breeders. In general, zebra finches exhibit no pronounced size dimorphism between the sexes [31]. However, we found females to be more vulnerable to differences in diet quality, which is in line with previous studies [32], [33]. Despite uncertainty about the underlying mechanisms, low female fledgling mass can result in lower female fecundity and/or survival [5], [34], [35]. Such sex-specific effects may carry indirect effects on males since competition for high quality females could also affect selection strength on males.

The compensation in biometry revealed substantial plasticity in timing of growth, which may represent costs to be paid later in life [15], [16]. Despite the lack of any long-term effects on the measured parameters in this study and on adult resting metabolic rate using the same subjects as here [15], [16], Krause et al. (2009) found a significant effect of early nutritional treatment on adult body mass loss after a short period of food deprivation. Subjects with a history of low quality nutrition as nestlings (LH) lost significantly more weight than did subjects that had been raised under better nutritional conditions (HH).

The dietary restrictions induced an elevation in baseline corticosterone in nestlings, shortly before fledging at day 17 and at the end of the fledgling period at day 35 (analysis on samples within detection limits). These findings show an empirical relationship between nutritional quality and a correlate for allostatic load [20], or wear and tear [21] during both the nestling and fledgling period. The elevated corticosterone levels at the end of both, the nestlings and the fledgling period in subjects that experienced poor nutritional conditions is particularly interesting as subjects had almost reached their growth asymptote at the beginning of the fledgling period [3]. Therefore, the negative impact of a low quality diet cannot be solely explained by their obvious effects during a period of substantial growth. Additionally, our results show that even if different developmental constraints (e.g. social or nutritional conditions, parasite infections) show similar effects [4], [11], [36], the physiological mechanisms and/or implications can be different and may not necessarily result in elevated corticosterone levels [27], [37]. In a previous study, Spencer et al. (2003) found an effect of low quality diet on biometry but not on corticosterone. This may be explained by qualitative (the low quality group received protein rich supplementation) and quantitative (1∶3 seed:husk ratio of a daily seed ration) differences between the applied nutritional treatments of the Spencer et al. (2003) and our study. However, it may also be important to consider domestication and its implications on the HPA axis, when comparing results of different studies and species. Previous studies indicate a decreased reactivity of the HPA-axis in domesticated compared to wild subjects [38], [39]. Thus, the difference between the Spencer et al. (2003) and our study may be partially due to the different history of the study subjects: our subjects were descendants (F9, for genetics see [40]) from wild caught zebra finches whereas the birds of Spencer et al. (2003) had been in captivity for many more generations.

Catch-up growth has been hypothesized to be demanding [15], [41], [42] but our results show that this effect cannot be easily translated to the physiological level. Catch-up growth, which may be as demanding as a steep growth rate earlier in life, did not result in higher corticosterone levels even though one may speculate that corticosterone would increase under less optimal nutritional conditions than applied in this study. Given that high levels of corticosterone can suppress growth [37], [43]–[46], our results suggest that such a regulatory mechanism operates on a relative rather than an absolute level. Independent of nutritional treatment, corticosterone titres were overall higher at day 17 than at day 35, when both groups went through a pronounced growth phase.

Taken together our results indicate that elevated levels of corticosterone may be only one of the driving mechanisms leading to short and long term effects of early nutritional conditions, although they may affect sensitivity to stress later in life [43].

The high levels of corticosterone at day 17 may mirror the transition from nestling to fledgling as corticosterone could ensure energy mobilization for enhanced motor activity and may facilitate rapid learning and memory formation during the period when offspring leave the nest and explore the environment [22]. The overall decrease in baseline corticosterone between day 17 and day 35 as well as the treatment effects indicate that the variation in corticosterone baseline titres was associated with the developmental phase and with the nature of the stressor [26], [27]. The positive relationship between order of sampling and circulating corticosterone at day 17 indicates the beginning of a typical adrenocortic stress response due to the varying handling time for different positions within the sampling sequence [30], [47]. Our data support a prediction of the developmental hypothesis, which suggests that altricial nestlings gradually increase their glucocorticoid stress response during the nestling stage until exhibiting an adult like profile [48].

Compensation for unfavourable early conditions, as shown here, may entail future costs [1], [6], [16], [41]. However, our analysis failed to detect an effect on plumage ornaments and on survival even though fewer individuals survived when they had received low quality food as nestlings (LH). Despite condition at fledging being a prominent predictor for survival in the wild [5], [34], survival within the laboratory cannot be transferred to natural conditions. Our findings thus rather reveal a potential for phenotypic plasticity present in a species as essential pre-requisite to adapt to ecological conditions. The lack of nutritional effects on male cheek development underlines that timing and duration of dietary restrictions matter. Cheek ornamentation is melanin-based containing 99.2% phaemelanin and 0.8% eumelanin [49] and even though its synthesis was recently shown to be affected by dietary manipulations [50] it is considered to be energetically cheap [51]. Our findings expand on these studies by showing that the expression of plumage ornaments is not affected by dietary history, whereas nutritional condition during the actual development of the ornaments affects their expression [52]. Thus, these ornaments provide information about environmental conditions encountered during a specific period. The tendency of males raised on a high quality diet throughout their first month (HH) to show more variability in plumage ornaments than birds that received a high quality diet only during either the nestling or fledgling period may be taken to suggest that exploiting ones full genotypic potential occurs only under optimal conditions. If females attend less to such signals when they have information on poor conditions during early development [53], [54], remains an interesting possibility to be shown.

To conclude, even a moderate shift within the limited optimal time window for reproduction can carry costs which may increase selection pressure on the young. Phenotypic plasticity appears to provide a mechanism to compensate for the effects of transient nutritional constraints. Elevated corticosterone provided a physiological correlate for nutritional diet but not for catch-up growth, suggesting catch-up growth to have rather future than current costs. Such an immediate physiological response may be a mechanism contributing to long term effects, which can resurface under stressful conditions in adulthood [16]. Thus, implications of nutritional constraints may have played a role in the evolution of strategies to refrain from initiating reproduction too early within the season.

## Materials and Methods

### Ethics Statement

The research was carried out according to the German laws for experimentation with animals (§ 8 Abs. 1 TierSchG, .V.m. § 2 Abs. 1.1 TierSchZustV NW 26.9.1989 (GV NW S. 508) and permission (I/04) to conduct the experiments was granted by the local authorities (Bezirksregierung Detmold; 4.10.2004).

### Subjects

Zebra finches of wild Australian origin from the laboratory colony in Bielefeld were allowed to breed under standardized conditions (July to December 2005). Females were transferred from their aviaries to separate breeding cages (83×30×39.5 cm) and allowed to acclimatize for two days. Then, males were randomly assigned to females controlling for relatedness for at least 2 generations. A nest box (12.5×12×14 cm) and nesting material were provided two days later. Pairs received a standard seed mix, hanging millet and water ad libitum and additionally a mixture of germinated seeds and commercial egg food (CéDé, Evergem, Belgium) daily. Greens were provided once weekly. Room temperature was 23° to 26°C with a 14∶10 light:dark regime with half an hour of dusk and dawn. Nests were checked daily between 0900 and 1100 hours to mark newly hatched chicks by cutting their down feathers. Out of 86 pairs, 36 pairs produced 149 hatchlings (day 0) from which 35 pairs raised 108 fledglings (day 17), from which 33 pairs raised 96 offspring until independence (day 35). Many pairs were first time breeders and the proportion of successful broods was similar to previous experiments.

### Experimental treatment and housing

All cages were assigned alternating to one of two diets once the oldest chick was 3 days old. Diets differed mainly in protein availability (Table 2). The minimum energy requirement for growing passerines (granivorous) is five times that of the adult birds and chicks require a protein level of 15–20% (as opposed to an adult protein requirement of 10–14%) [55], [56]. The low quality (L-) diet (protein content 10.9%, Table 2) consisted of ad libitum dried seed mix and water. Two times weekly, water was enriched with vitamins (Veyx-Pharma, BioWeyxin - Multi-Mulgat). The high quality (H-) diet (protein content 8.2–16.2%, Table 2) additionally comprised millet, a mixture of germinated seeds and commercial egg food (Cédé) daily and two times weekly grated salads, greens, fruits and vegetables were provided. Breeding pairs received the two diets in different order to simulate three natural scenarios: breeding earlier than optimal (LH), breeding later than optimal (HL) and as a positive reference for optimal timing (HH). The low-high (LH) group received a L-diet from day 3 until day 17 and a H-diet from day 17 until day 35. The high-low (HL) group received a H-diet from day 3 until day 17 and a L-diet from day 17 until day 35. The high-high (HH) group received a H-diet from day 3 until day 35 (Fig. 1). At nutritional independence (day 35 of the youngest of a brood), offspring were transferred to mixed groups where all received a standard diet (intermediate between the L- and H-diet). Distribution of offspring (excluded deaths before day 4) across treatments: LH = 45, HL = 43 and HH = 38.

**Table 2:** Dietary contents [%] of four different food sources used within the nutritional treatment.

	Seed mix	Germinated seeds	Egg food	Millet
**Crude protein**	10.90	8.20	16.20	11.30
**Crude fat**	4.13	3.05	5.00	4.30
**Crude fiber**	9.15	6.60	4.20	7.00
**Moisture**	9.41	31.88	11.00	10.00
**Crude ash**	2.98	2.03	5.00	3.00
**Diet**	L, H	H	H	H

Food sources included within the low (L) and the high (H) quality treatment are indicated in the row diet.

### Biometric and corticosterone measurements

Biometric measurements were taken at the age of 0, 17, 35, 65, and 280. Blood samples were taken from the brachial vein within 3 min after chick removal from the nest at day 17 (mean age of a brood) and 35 (last hatched chick of a brood). The position of a subject within the sampling sequence of the whole nest and the exact duration of sampling [s] was recorded for each individual. Samples were stored on ice immediately and centrifuged at ∼6000 g at 4°C for 10 min. 1 µl of separated plasma was taken for sexing [57] and the rest was stored at −70°C until hormone assay.

Corticosterone concentration was determined by direct radioimmunoassay (RIA), following the procedures described in [58]. Mean ± s.d. extraction efficiency for plasma corticosterone was 89.0±1.2%. The lower detection limit of the corticosterone assay was determined as the first value outside the 95% confidence intervals for the zero standard (B_max_) and was 5.9 pg/tube (day 17) and 7.3 pg/tube (day 35). Intra-assay coefficients of variation were 1.3% (day 17) and 3.5% (day 35). The inter-assay variation was 5.1%. If a sample concentration was below detection limit its value was set at the respective detection value.

### Plumage measurements

To compare male cheek patch development, subjects were photographed at day 65 (mean; range 60–73 days) against a black board with a digital camera (Canon Ixus30, Kodak LS753). Photographs were taken under standard light conditions from a standard distance. The pigmented cheek area and the shape of the beak were determined by using the “magic wand” tool in Adobe Photoshop 7.0 (Adobe Systems Inc. San Jose, CA, USA) [52], [59]. To obtain standardized measurements for each bird and image, the ratio of mean cheek patch to mean beak was calculated (n = 44 males; with adequate photos). For each picture, the pixels of cheek patch and beak were taken three times and for each individual at least two different pictures were analyzed.

### Statistical analysis

Data were analyzed by using the free software R (R Development Core Team, 2006). Linear mixed effect (LME) models [60] were calculated for each response variable (body mass, wing length, tarsus length and corticosterone concentration) with fixed effects: treatment, sex and brood size. Predictions for random effects were made for the exact age at sampling and the nest. For the corticosterone analysis, sequence of blood sampling and individual sampling time was additionally included as fixed effects. The most parsimonious model was selected by stepwise deletion of non significant terms from the maximum model by using Akaike's Information Criterion. Data were transformed accordingly when model residuals violated normal distribution (Shapiro-Wilk normality test). If normality assumptions were not met after transformation non-parametric tests were applied. Given that at day 35 a high proportion of samples was below the detection limit, which can mask present effects, corticosterone concentrations at day 35 were further analyzed with a separate LME using only samples within assay's detection limits. Variance in cheek patch expression between the treatment groups was tested with Fisher F-Test for variance homogeneity. Treatment effects on number of deaths (until November 2009, considering subjects surviving ≥4 days) was analyzed with Pearson's Chi^2^-test on Contingency tables. Survival estimates (Kaplan-Meier) were extracted for each treatment over the course of 1200 days (censoring) and graphically represented (Fig. 4). Sample sizes in the statistical tests differ, as for some individuals not all measurements were available (see criterion about inclusion into analysis). Subjects were only included within the analysis when they survived at least seven days post treatment (except analysis on number of deaths and survival estimates).

## Supporting Information

Table S1 Raw means ± s.e. for all biometric measurements and timepoints. Until day 17 subjects fed either on a low quality (L) or high quality (H) diet. Post day 17 subjects belonged to either LH  =  low-high (L as nestlings), HL  =  high-low (L as fledglings) or HH  =  high-high (H throughout) treatment. N  =  number of nests n  =  subjects came from.
(0.03 MB XLS)
